# Exploring the Potential of Extracellular Vesicles from Atlantic Cod (*Gadus morhua* L.) Serum and Mucus for Wound Healing In Vitro

**DOI:** 10.3390/biology14070870

**Published:** 2025-07-17

**Authors:** Stefania D’Alessio, Igor Kraev, Bergljót Magnadóttir, Sigrun Lange

**Affiliations:** 1 Pathobiology and Extracellular Vesicles Research Group, School of Life Sciences, University of Westminster, 115 New Cavendish Street, London W1W 6UW, UK; s.dalessio@westminster.ac.uk; 2 Electron Microscopy Suite, Faculty of Science, Technology, Engineering and Mathematics, Open University, Milton Keynes MK7 6AA, UK; igor.kraev@open.ac.uk; 3 Institute for Experimental Pathology, University of Iceland, Keldnavegur 3, 112 Reykjavik, Iceland; bergmagn@hi.is

**Keywords:** Atlantic cod (*Gadus morhua* L.), extracellular vesicles, tissue regeneration, wound healing, FGF, vimentin, proteome, KEGG, gene ontology

## Abstract

Novel therapeutic approaches for wound healing have included biomaterials from the Atlantic cod (*Gadus morhua* L.), with promising results in wound management. The use of extracellular vesicles (EVs), which can be isolated from cod biofluids, remains to be studied. EVs play key roles in cellular communication, and their use both as biomarkers and as therapeutic agents is widely reported in human pathologies, particularly with respect to mesenchymal stem cells. This pilot study characterized the total proteomic cargo content of EVs from cod serum and mucus and assessed the EVs’ potential for regenerative activity in wound-healing processes, using human and mouse fibroblast and keratinocyte in vitro scratch injury models. The pro-regenerative potential of both cod serum EVs and mucus EVs was identified, with differing capacities for accelerating wound closure in fibroblast and keratinocyte cells. This was further supported by varying effects of the cod serum EVs and mucus EVs on cellular vimentin and FGF-2 levels. The serum EV and mucus EV protein cargoes differed with respect to abundance of protein hits and associated enriched functional GO and KEGG pathways, but both were associated with immune, stress and wound-healing processes. Cod EVs may present as innovative therapeutic options for regenerative medicine applications, and our reported findings provide valuable insights for future in-depth studies.

## Simple Summary

Skin wound healing is a major research field in medicine, where improved and new therapeutic approaches are in demand. Biological agents and materials from the Atlantic cod have shown promising potential in wound treatment applications. Therefore, it is important to understand both cellular and molecular factors involved and explore further cod-derived agents. Extracellular vesicles (EVs) are small membrane vesicles released from cells and can be isolated from biofluids, such as blood and mucus. EVs are important for cell communication, and EVs from various cell types, including stem cells, have been identified as potent agents for tissue regeneration. This is the first study to assess the possible pro-regenerative effects of EVs isolated from cod mucus and serum in wound healing models of human and mouse cells. We report preliminary promising results on the protective effects of cod EVs in wound healing, with the potential to accelerate wound closure and elevate key factors involved in promoting wound healing.

## 1. Introduction

Wound healing research relies on various cellular in vitro and in vivo animal models, including mice, rats, rabbits, guinea pigs, pigs, and zebrafish, to elucidate mechanisms and processes involved in tissue regeneration [1]. There is increased interest in the identification and application of new agents in wound healing, including products derived from fish, such as the Atlantic cod (*Gadus morhua* L.). Cod collagen has for example been identified as an innovative, sustainable, and efficacious product for cosmetic and biomedical applications [2,3], in tissue regeneration and in drug delivery [4]. Cod trypsin has been assessed for use in biomedicine, including infection [5]. Formulations of cod liver oil have been investigated for diabetic wound healing in murine in vivo models, showing significantly reduced wound areas and improved healing [6], while skin xenografts from cod have shown great promise in acute wound healing [7]. Cod skin exhibits a unique molecular composition, including high Omega-3 polyunsaturated fatty acids (PUFAs), which may partly contribute to promoting wound healing, including burn wounds [8,9]. As cod-derived products have shown promising results in the context of tissue regeneration, it is of considerable interest to identify further cellular and molecular factors involved.

Extracellular vesicles (EVs) are important messengers in cellular communication and carry various cargoes, including nucleic acids, non-coding RNAs and proteins. EVs from various sources have been explored as potential therapeutic agents in regenerative medicine, and it is therefore of great interest to investigate whether cod-derived EVs isolated from biological fluids, including from serum and mucus [10,11,12], may affect wound healing. Such alternative sources of EVs may be relevant for regenerative medicine, as to date, the largest body of research has been on mesenchymal stem cell (MSC)-derived EVs in a range of in vivo and in vitro models [13,14,15,16,17,18,19]. In comparison, only a limited number of studies have been carried out on EVs derived from biofluids of diverse species across phylogeny [20], and from plants and animal derivates, such as aloe vera or royal jelly [21,22].

This pilot study aimed to assess the potential of cod serum EVs and mucus EVs in wound healing, using cellular in vitro scratch injury models of three cell lines: (1) mouse fibroblasts (3T3.L1), (2) human keratinocytes (HaCaT), and (3) human dermal fibroblasts (HDFa). Following cod EV application, wound closure was assessed at different time points, up to 24 h post injury. The total proteomic content of the EVs was furthermore analysed for the identification of pro-regenerative factors. In addition, fibroblast growth factor 2 (FGF-2), which is implicated in wound-healing processes including cell migration [23,24], and vimentin, an intermediate filament marker of fibroblasts and wound healing [25,26], were assessed by immunocytochemistry, following cod EV application in the HDFa model.

## 2. Materials and Methods

### 2.1. Cod Serum and Mucus Extracellular Vesicle Preparation and Characterisation

Cod serum EVs and mucus EVs were isolated from cod serum and mucus, respectively, collected from experimentally farmed adult cod (*Gadus morhua* L.) at the Marine Institute’s Experimental Fish farm Staður, Grindavik, Iceland (under license from the Institute for Experimental Pathology, University of Iceland, number #0002 kt-650269—4549, approved by the central animal ethics committee in Iceland; Icelandic Food Regulation Authority, MAST Mavælastofnun). The EVs were isolated according to our previously standardised and described protocols [10,11,12,27], using sequential centrifugation and ultracentrifugation, adhering to recommendations of MISEV [28]. In brief, cod serum and mucus aliquots (pool of n = 10 per preparation) were diluted 1:5 in Dulbecco’s PBS (DPBS, ultrafiltered using a 0.22 µm filter before use) and centrifuged for 20 min at 4000× *g* at 4 °C to remove apoptotic bodies and aggregates before collecting the supernatant, which was ultra-centrifuged at 100,000× *g* at 4 °C for 1 h. The EV-enriched pellets were resuspended in 500 µL DPBS for washing and ultra-centrifuged again for 1 h at 100,000× *g* at 4 °C. The final EV pellets were resuspended each in 50 µL of DPBS and stored at −80 °C for further experiments and analysis.

EVs were quantified and profiled by nanoparticle tracking analysis (NTA), which involved diluting concentrated EV pellets 1/1000 in DPBS before application to the NanoSight NS300 (Malvern Panalytical Ltd., Malvern, UK) using the 488 nm blue laser. Four 60 sec recordings at camera setting level 13 with post-analysis level 3 were used to generate replicate histograms (NanoSight software 3.0, Malvern Panalytical Ltd.), showing means with confidence intervals.

Western blotting was used to assess two EV surface markers, CD63 (ab216130, Abcam, Cambridge, UK) and Flotillin-1 (ab41927, Abcam), as previously described [11,12]. Protein separation was carried out using 4–20% gradient TGX gels (BioRad, Watford, UK) followed by Western blotting using a Trans-Blot^®^ SD semi-dry transfer cell (BioRad, UK). Membranes were blocked with 5% bovine serum albumin (BSA, Sigma-Aldrich, Dorset, UK) in Tris-buffered saline (TBS) containing 0.1% Tween20 (BioRad, UK; TBS-T) for 1 h at room temperature (RT), followed by primary antibody incubation overnight at 4 °C, washing in TBS-T and incubation at RT for 1 h with HRP-conjugated anti-rabbit IgG (BioRad, diluted 1/3000 in TBS-T). The membranes were washed in TBS-T and visualized digitally using enhanced chemiluminescence (ECL, Amersham, UK) and a UVP BioDoc-ITTM System (Thermo Fisher Scientific, Dartford, UK).

For EV imaging by transmission electron microscopy (TEM), EVs were fixed with 2.5% glutaraldehyde in 100 mM sodium cacodylate buffer (pH 7.0) for 1 h at 4 °C, re-suspended in 100 mM sodium cacodylate buffer (pH 7.0), placed on to a grid with a glow discharged carbon support film, and stained with 2% aqueous Uranyl Acetate. The JEOL JEM 1400 transmission electron microscope (JEOL, Tokyo, Japan) was used, operated at 80 kV at a magnification of 30,000× to 60,000×. Digital images were recorded using an AMT XR60 CCD camera (Deben, Bury St. Edmunds, UK).

### 2.2. EV Protein Cargo Analysis by Liquid Chromatography with Tandem Mass Spectrometry (LC-MS/MS); Protein–Protein Interaction Network and Pathway Enrichment Analysis

To identify the protein cargoes of cod serum EVs and mucus EVs, LC–MS/MS was carried out following in-gel digestion using a Dionex Ultimate 3000 RSLC nanoUPLC system (Thermo Fisher Scientific Inc., Waltham, MA, USA), in conjunction with a QExactive Orbitrap mass spectrometer (Thermo Fisher Scientific Inc., Waltham, MA, USA). The data were processed post-run using Protein Discoverer (version 2.1., Thermo Scientific). All MS/MS data were converted to mgf files, which were submitted to the Mascot search algorithm (Matrix Science, London, UK) to identify protein hits. A protein hit search identified from cod serum EVs and mucus EVs was conducted against the species-specific *Gadus morhua* database (Gadus_morhua_20190405; 1283 sequences; 308,668 residues), with the significance threshold set at *p* < 0.05 and the cut-off ion score at 16, or against the Teleostei UniProt database (CCP_Teleostei Teleostei_20201009; 4,085,639 sequences; 2,121,030,378 residues), with the significance threshold set at *p* < 0.05 and the cut-off ion score at 53 (Cambridge Proteomics, University of Cambridge, UK). Protein–protein interaction networks were generated by feeding protein hits for each EV group into the STRING database (https://string-db.org/; accessed on 20 June 2025) and analysed based on the *Homo sapiens* database, with settings at medium confidence. The protein–protein interaction networks were assessed for enrichment of gene ontology (GO), KEGG and reactome pathways, with files exported as jpeg images.

### 2.3. In Vitro Application of Atlantic Cod Serum EVs and Mucus EVs on In Vitro Wound Healing Models

Three cell lines were used in this study for the assessment of cod EVs (either serum EVs or mucus EVs or both) on in vitro scratch injury models, mimicking wound healing. In the first instance, the mouse fibroblast (3T3-L1) cell line was used to assess the potential of cod serum EVs for accelerating wound healing closure. Next, cod serum EVs’ potential for keratinocyte wound closure was assessed using the human keratinocyte HaCaT cell line. Due to more availability of cod serum than cod mucus for this pilot study, only cod serum EVs were assessed in 3T3-L1 and HaCaT cells. Thereafter, the human fibroblast HDFa cell line was used for the assessment of both cod serum EVs and mucus EVs on accelerating scratch injury closure. Changes in FGF-2 and vimentin levels in response to EV application were in addition assessed in the HDFa scratch injury model.

#### 2.3.1. Mouse Fibroblast (3T3-L1) Scratch Injury Model

Mouse fibroblast cells (3T3-L1, ATCC) were cultured in a T75 flask (Thermo Fisher Scientific, Cheshire, UK) in 1× DMEM (Gibco™, London, UK) containing 10% FBS (MP Biomedical Fetal Bovine Serum, Fisher Scientific) and 1% penicillin–streptomycin (Gibco™) until 70–80% confluency. Cells were trypsinized with 3 mL of 0.25% trypsin-EDTA (Gibco™), incubated for 10 min, and thereafter neutralised with 5 mL of DMEM and spun down at 1000× *g* for 5 min. The cell pellet was resuspended in 1 mL DMEM and plated in a 12-well plate (Thermo Fisher Scientific, Cheshire, UK) at a concentration of 2 × 10^2^ cells/well and cultured in complete medium (DMEM) containing 10% FBS and 1% penicillin–streptomycin (Thermo Fisher Scientific) until 90% confluency was reached (24–36 h) to obtain a full fibroblast monolayer. A linear scratch was performed using a sterile 200 µL plastic pipette tip. Any cellular debris was removed by washing the wells with phosphate-buffered saline (PBS). Fresh DMEM medium was added to a set of three wells (control group), and isolated EVs from 1 mL Atlantic cod serum, containing approximately 1.55 × 10^10^ +/− 9.17 ×10^8^ particles/mL EVs, were diluted in 1 mL DMEM and added to a set of three wells per treatment group. Images were taken using EVOS FL Auto Imaging Systems (Thermo Fisher Scientific Inc., UK) at time points of 0 h, 6 h and 24 h, following the optimisation of assessment of the wound closure time using the 4× objective. For the assessment of wound healing closure, ImageJ software (ij154) (https://imagej.nih.gov/ij/) was used, measuring the area of scratch volume with the freehand selection tool. Histograms were generated to compare differences in gap closure size comparing the EV-treated versus the control (medium only) scratch injuries using GraphPad Prism (version 10) and one-way ANOVA, with significance considered at *p* < 0.05.

#### 2.3.2. Human Keratinocytes (HaCaT) Scratch Injury Model

Human immortalised keratinocytes (HaCaT; 300493, Cytion, Eddelheim, Germany) were assessed by scratch wound assay, using the same approach as described in Section 2.3.1. After reaching 80% confluency, the cells were trypsinized with 3 mL of 0.25% trypsin-EDTA, incubated for 7–10 min, and thereafter neutralised with 5 mL of DMEM and spun down at 1000× *g* for 5 min. Cell pellets were resuspended in 1 mL DMEM, seeded into 12-well plates (Thermo Fisher Scientific) at a concentration of 2 × 10^2^ cells/well and cultured in media containing 10%FBS and 1% penicillin–streptomycin to nearly confluent cell monolayers for about 24 h to 36 h, in order to obtain 80% confluency and conduct scratch assay analysis, which was performed as described in Section 2.3.1 (applying cod serum EVs compared to controls (medium only)). Images were taken using EVOS FL Auto Imaging Systems at time points of 0 h and 24 h, following the optimisation of assessment of the wound closure time, using the 4× objective.

#### 2.3.3. Human Dermal Fibroblasts (HDFa) Scratch Injury Model

Human adult dermal fibroblasts (HDFa) (Cat. No. C-013-5C; Gibco™) were grown to 80% confluency. Cells were trypsinised with 3 mL of 0.25% trypsin-EDTA, incubated for 2–3 min, neutralised with 5 mL of DMEM, and spun down at 1000× *g* for 5 min. Cell pellets were resuspended in 1 mL DMEM, and the cells were seeded into a 12-well plate (ThermoFisher) at a concentration of 2 × 10^2^ cell/well and cultured in media containing 10% FBS and 1% penicillin–streptomycin to nearly confluent cell monolayers in order to obtain 80% confluency. The scratch assay was performed as described in Section 2.3.1. For the HDFa cells, both serum EVs (1.55 × 10^10^ +/− 9.17 × 10^8^ particles/mL EVs, diluted in 1 mL DMEM and added to a set of three wells per treatment group) and mucus EVs (1.29 × 10^10^ +/− 1.22 × 10^9^, diluted in 1 mL DMEM and added to a set of three wells per treatment group) were assessed and compared with control wells treated with medium only. Images were taken using EVOS FL Auto Imaging Systems at time points of either 0 h versus 6 h, or 0 h versus 24 h for cod mucus EV and serum EV application, respectively, following the optimisation of assessment of the wound closure time using the 4× objective.

### 2.4. Immunocytochemistry for FGF-2 and Vimentin Staining on HDFa Cells Following Cod EV Treatment

HDFa cells were seeded onto a 12-well plate at a concentration of 3 × 10^2^ cells/well and cultured until 80% confluency was reached, and scratch injury was carried out as described above (Section 2.3.1), applying either the cod serum EVs or mucus EVs post scratch. For ICC, media was removed, and the cells were rinsed with PBS-T (1× PBS + 0.1% Tween20) and fixed at room temperature for 10 min with 4% paraformaldehyde in PBS (pH 7.4) (Thermo Fisher Scientific). The wells were then washed three times with ice-cold PBS, incubated for 10 min with PBS containing 0.2% Triton X-100 (Sigma Aldrich, Dorset, UK) for permeabilization, and rinsed with PBS three times for 5 min. Blocking (1% BSA + 22.52 mg/mL glycine in PBST) was carried out for 30 min at RT followed by incubation with Anti-Vimentin (ab92547, Abcam, Cambridge, UK) and Anti-FGF2 (ab208687, Abcam) antibodies, diluted 1/500 in 1% BSA + PBS-T overnight at 4 °C. The cells were washed for 3 × 5 min in PBS before incubation in secondary antibody (Goat Anti-Rabbit IgG Alexa Fluor^®^ 488 or Alexa Fluor^®^ 647, Abcam, diluted 1/1000) in 1% BSA for 1 h at room temperature in the dark, then washed 3 × 5 min with PBS and incubated for 1 min with 0.1–1 µg/mL of DAPI solution (Cat no. 62248, Thermo Fischer Scientific), followed by rinsing with PBS. FGF-2 and vimentin staining were visualised using EVOS FL Auto Imaging Systems, using the 4× objective. For FGF-2 staining, images were obtained at 8 h and 24 h timepoints following scratch injury, comparing serum EV or mucus EV application. For vimentin staining, images were obtained at 8 h post scratch, following mucus EV application. Fluorescence images were quantified using ImageJ and measurements imported to GraphPad Prism (version 10) for statistical analysis.

### 2.5. Statistical Analysis

For comparison of datasets between groups, GraphPad Prism version 10 was used, applying one-way ANOVA for comparison between groups, with significance regarded at *p* < 0.05. STRING analysis was carried out with medium confidence (https://string-db.org/, accessed on 20 June 2025).

## 3. Results

### 3.1. Cod Serum EV and Mucus EV Characterisation

**Figure 1:**
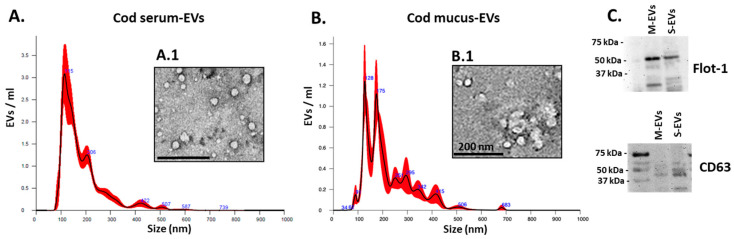
Cod serum EV and mucus EV characterization. (**A**) Cod serum EVs; a representative NTA histogram is shown (the black line represents the mean, and the red line represents the standard error for the mean, SEM); EV imaging by TEM is shown in (**A.1**) (scale bar = 200 nm). (**B**) Cod mucus EVs; a representative NTA histogram is shown, and EV imaging by TEM is shown in (**B.1**). (scale bar = 200 nm) (**C**) Western blotting for two EV-specific markers: CD63 and Flot-1 on mucus EVs (M-EVs) and serum EVs (S-EVs).

For serum EVs, the NTA analysis showed a poly-dispersed population in the size range of 60–500 nm with peaks at 115 and 206 nm (Figure 1A; TEM images of serum EVs are shown in Figure 1A.1). For the mucus EVs, the NTA analysis shows a poly-dispersed population in the size range of 35–500 nm, with peaks at 90, 128, 175 and 295 nm (Figure 1B; TEM images of mucus EVs are shown in Figure 1B.1). The concentration of EVs was determined based on the NTA quantification of particles per mL and estimated at 1.55 *×* 10^10^ +/− 9.17 *×* 10^8^ particles/mL EVs per 1 mL cod serum and 1.29 *×* 10^10^ +/− 1.22 *×* 10^9^ particles/mL in EVs per 1 mL of cod mucus, respectively. Western blotting confirmed the positive detection of CD63 and flotillin-1 (flot-1) EV surface markers for both mucus EVs and serum EVs (Figure 1C). This EV characterisation corresponds to our previous reports on EVs isolated from cod mucus and serum [10,11,12].

### 3.2. LC-MS/MS Analysis of Cod Serum EVs and Mucus EV Protein Cargoes

The total protein cargo content of the cod serum EVs and mucus EVs was carried out by LC-MS/MS, with protein hits assessed against the Atlantic cod UniProt database (CCP_*Gadus*_*morhua*_20190405; 1283 sequences: 308,668 residues) and the teleost UniProt database (CCP_Teleostei Teleostei_20201009; 4,085,639 sequences; 2,121,030,378 residues). Protein hits for cod serum EVs are provided in Supplementary Table S1 and for cod mucus EVs in Supplementary Table S2. In the serum EVs, 23 protein hits were identified. In the mucus EVs, 57 protein hits were identified, of which 8 hits were common with the serum EV proteome (highlighted in yellow): fast skeletal muscle alpha-actin, beta-actin, serotransferrin, 60 s ribosomal protein L22, Galectin, Elongation factor 1 alpha, Profilin, and Ribosomal protein L15.

### 3.3. Protein–Protein Interaction Network and Pathway Enrichment Analysis of Cod Serum EV and Mucus EV Proteomes

**Figure 2:**
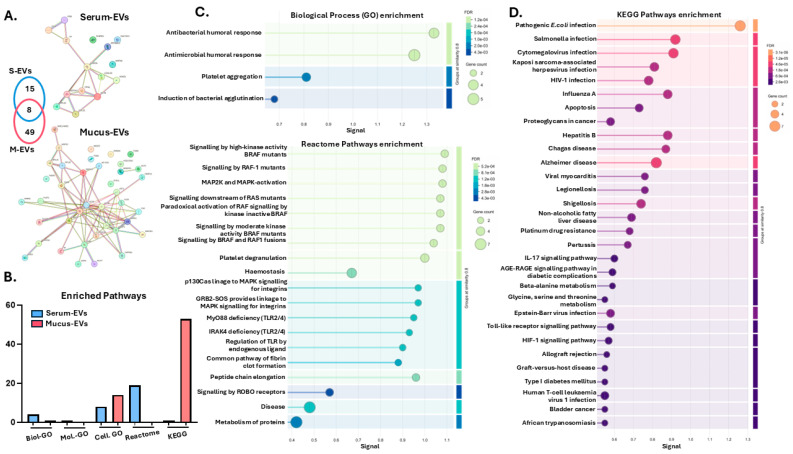
Protein cargo analysis of cod serum EVs and mucus EVs. (**A**) Protein–protein interaction networks for serum EV (S-EV) and mucus EV (M-EV) proteomes, showing shared and unique protein hits in the Venn diagram. (**B**) Summary of numbers of enriched GO, reactome and KEGG pathways associated with the serum EV and mucus EV proteomes. (**C**) Biological and reactome pathways associated with the cod serum EV proteome. (**D**) Top 30 KEGG pathways associated with the cod mucus EV proteome.

Protein–protein interaction networks were generated for the cod serum EV and mucus EVs proteomes, respectively (Figure 2A). Pathway enrichment analysis was carried out for gene ontology (GO), KEGG and reactome pathways (Figure 2B). There were considerable differences observed between the EV proteomes, with 4 biological process GO pathways (Figure 2C), 8 cellular component GO pathways (linked to blood microparticle, collagen-containing extracellular matrix, exosome and extracellular space), 1 KEGG pathway (platelet activation) and 19 reactome pathways associated with the serum EV proteomes (Figure 2C). For the mucus EV proteomes, 1 biological process GO pathway (organonitrogen compound metabolic process), 14 cellular component GO pathways (associated with MHC class I protein complex, death-inducing signalling complex, secretory granule, and caspase complex), and 53 KEGG pathways associated with various immune related functions were enriched (see Figure 2D for the top 30 KEGG pathways enriched, based on signal and false discovery rate (FDR)).

### 3.4. Application of Cod Serum EVs and Mucus EVs on Fibroblast and Keratinocyte In Vitro Wound Healing Scratch Assays

To assess the effectiveness of cod serum EVs or mucus EVs on wound healing in vitro, three different cell lines were used: mouse fibroblasts (3T3.L1), human keratinocytes (HaCaT), and human dermal fibroblasts (HDFa). Due to the greater availability of cod serum than mucus in this pilot study, cod serum EVs were assessed in all three cell lines, while cod mucus EVs were only assessed in the HDFa cells.

#### 3.4.1. Application of Cod Serum EVs on Mouse Fibroblast (3T3.L1) Scratch Assay

**Figure 3:**
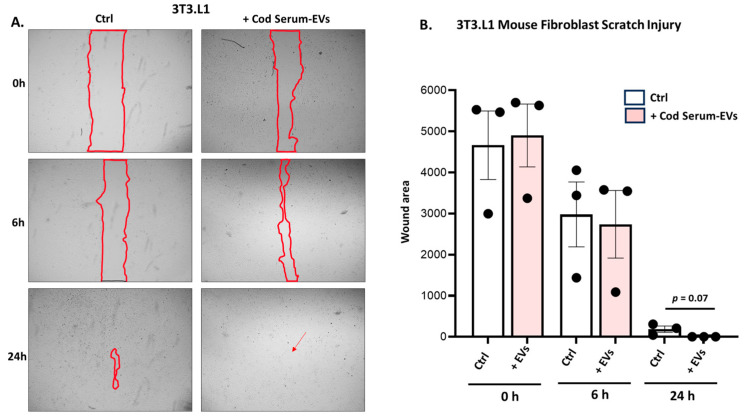
Assessment of cod serum EVs’ efficiency in promoting wound healing in 3T3.L1 mouse fibroblasts. (**A**) Representative images of mouse fibroblast (3T3-L1 cell line) scratch assay analysis of control group and cod serum EV-treated groups with gap closure (outlined with red) assessed at 6 h and 24 h. (**B**) Comparison of means between the control group (medium only) and cod serum EV treatment group at 0 h, 6 h, and 24 h. Data are represented as mean ± SEM from three independent experiments. Exact *p*-values are shown (n = 3 per group).

Scratch assays were performed on mouse fibroblast (3T3-L1 cell line) cells with cod serum EVs added (1.55 × 10^10^ +/− 9.17 × 10^8^ particles/mL), while control wells contained medium only. Images were taken at 0, 6 h, and 24 h. When comparing the control and EV-treated cells at 6 h post injury (Figure 3A), no statistically significant difference was observed; however, there was a trend suggesting a reduction in scratch volume. While not reaching statistical significance, a trend (*p* = 0.07) toward increased wound closure was detected for the-EV treated wells compared with the control (medium only)-treated wells at 24 h post scratch (Figure 3B).

#### 3.4.2. Application of Cod Serum EVs on Human Immortalized Keratinocyte (HaCat) Scratch Assays

**Figure 4:**
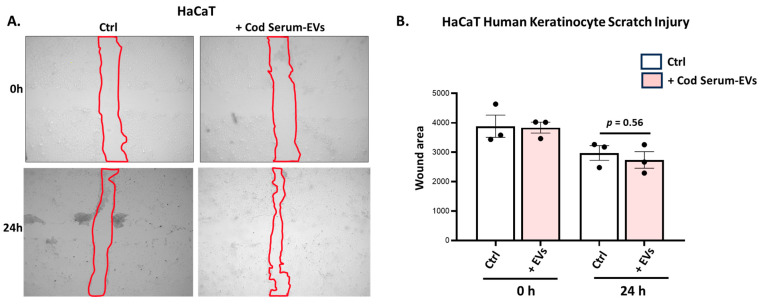
Assessment of cod serum EVs′ efficiency in promoting wound healing in HaCaT human keratinocytes. (**A**) Representative images for the HaCaT cell wound scratch assay analysis of control group and cod serum EV-treated groups with gap closure (outlined with red) assessed at 24 h. (**B**) Comparison of means between the control group (medium only) and cod serum EV treatment group at 0 h and 24 h. Data are represented as mean ± SEM from three independent experiments. Exact *p*-values are shown (n = 3 per group).

Scratch assays were performed on human immortalised keratinocyte (HaCaT) cells with cod serum EVs applied as described in Section 3.4.1, with control wells supplied with medium only. Pictures were obtained at 0 h and 24 h, with representative images shown in Figure 4A. A trend for increased gap closure was observed for the cod serum EV-treated wells; however, this was not statistically significant (*p* = 0.56) compared with the control group at 24 h (Figure 4B).

#### 3.4.3. Application of Cod Serum EVs and Mucus EVs on the Human Dermal Fibroblast (HDFa) Scratch Assay

**Figure 5:**
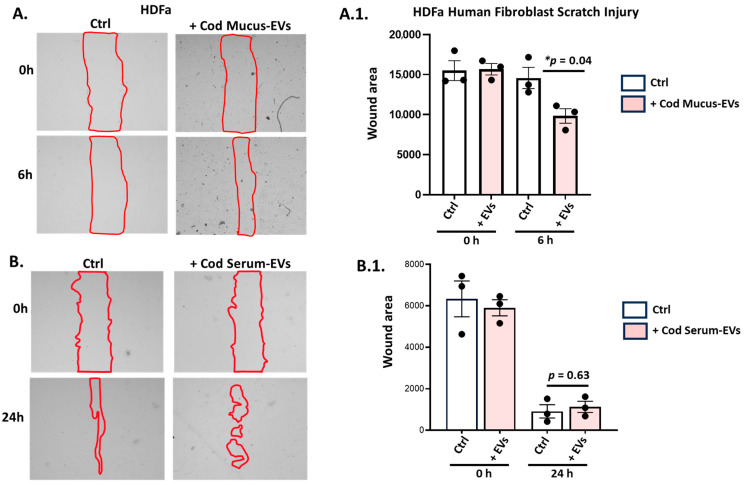
Assessment of cod mucus EVs’ and serum EVs’ efficiency in promoting wound healing in human dermal fibroblasts (HDFa). (**A**) Representative images of HDFa scratch assay analysis comparing control (medium only) and cod mucus EV-treated groups with gap closure (outlined with red) assessed at 6 h post scratch injury, with quantitative analysis of n = 3 presented in the histogram in (**A.1**). (**B**) Representative images of HDFa scratch assay analysis comparing control and cod serum EV-treated groups with gap closure assessed at 24 h, with quantitative analysis of n = 3 presented in the histogram in (**B.1**). Exact *p*-values are shown (* significance at *p* < 0.05).

Scratch assay was performed on human dermal fibroblasts (HDFa), followed by application of either cod mucus EVs (1.29 × 10^10^ +/− 1.22 × 10^9^ particles/mL) or serum EVs (1.55 × 10^10^ +/− 9.17 × 10^8^ particles/mL); control wells contained medium only. Images were taken at time points of 0 h and 6 h (for mucus EVs) or 24 h (serum EVs) (Figure 5A,B). HDFa cells treated with cod mucus EVs showed significant differences in gap closure after 6 h (Figure 5A.1.; *p* = 0.04), while no significant differences were observed for cod serum EVs 24 h post scratch injury (Figure 5B.1).

### 3.5. Effects of Cod Serum EVs and Mucus EVs on FGF-2 and Vimentin in the HDFa Scratch Injury Model

**Figure 6:**
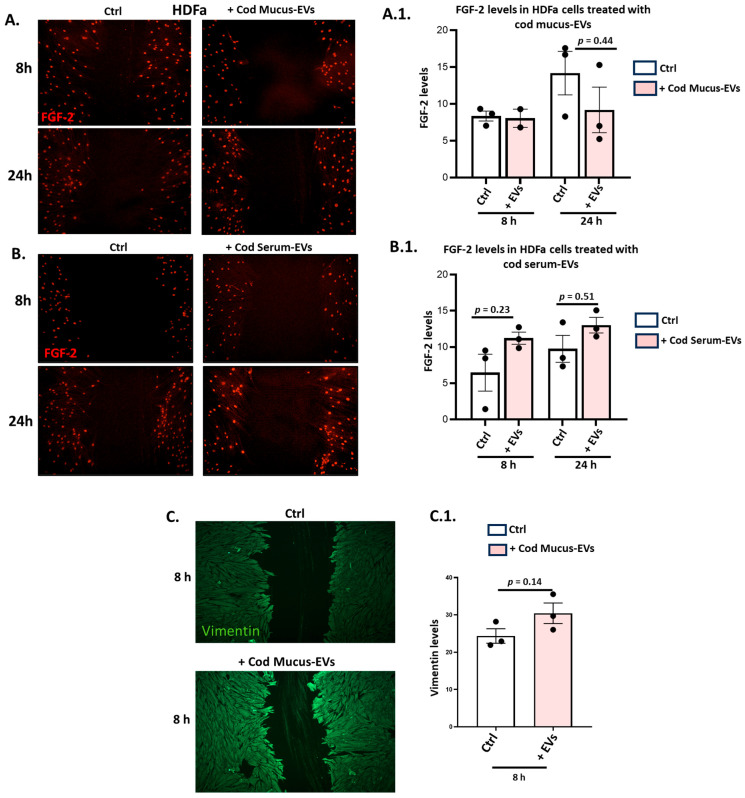
Assessment of FGF-2 and vimentin levels by immunocytochemistry in HDFa cells treated with cod mucus EVs or serum EVs. (**A**) Representative immunofluorescence images showing FGF-2 levels in HDFa cells treated with cod mucus EVs; (**A.1**) Histograms represent changes in fluorescence intensity of FGF-2 in mucus EV treated compared to control (medium only) cells at 8 h and 24 h post scratch injury. (**B**) FGF-2 levels in HDFa cells treated with cod serum EVs; (**B.1**) Histograms represent changes in fluorescence intensity of FGF-2 in serum EV treated, compared with control (medium only) treated cells at 8 h and 24 h post scratch injury. (**C**) Representative immunofluorescence images show Vimentin levels in HDFa cells treated with cod mucus EVs, compared with control (medium only) cells. (**C.1**) The histogram represents changes in fluorescence intensity of vimentin staining in mucus EV-treated, compared with control (untreated, medium only) cells at 8 h post scratch injury. Data are represented as mean ± SEM, exact *p*-values are shown (n = 3).

HDFa cells treated with cod serum EVs and mucus EVs were assessed for changes in anti-FGF-2 and anti-vimentin staining by immunocytochemistry. FGF-2 staining did not change significantly in response to cod mucus EV treatment (8 h and 24 h timepoints) (see representative images in Figure 6A, with quantitative analysis in Figure 6A.1). Treatment with serum EVs did, on the other hand, show some trends towards increased FGF-2 levels at 8 h and 24 h post scratch injury, albeit they were not statistically significant (Figure 6B,B.1). The application of mucus EVs was furthermore assessed for effects on vimentin staining in the HDFa cells, showing a trend toward increased vimentin levels at 8 h post scratch injury (Figure 6C), albeit not reaching statistical significance (Figure 6C.1, *p* = 0.14).

## 4. Discussion

Extracellular vesicles (EVs) have received considerable interest in regenerative medicine, including in the context of wound healing applications. EVs are mediators of cellular communication, which through their selective cargo delivery of protein, lipids, and nucleic acids can modulate various physiological processes such as angiogenesis, inflammation, cell proliferation, and differentiation, thereby exerting profound effects on tissue repair mechanisms [29,30]. EVs derived from various human cell sources, such as stem cells (and in particular mesenchymal stem cells (MSCs)), platelets, dendritic cells, macrophages and epithelial cells, have been extensively studied regarding their functions and possible therapeutic properties in the field of regenerative medicine [16,19,31,32,33]. As standardisation protocols for many of these EV sources still face considerable challenges for therapeutic applications in the clinic [30,34,35], investigations into other potential EV sources have received increasing attention. This includes EVs derived from different biological sources such as milk [36,37,38,39], bees’ royal jelly [22], plants [21] and fruit [40,41]. In addition, EVs from Akoya pearl oyster mucus [20] and olive flounder plasma [42] were recently assessed for their regenerative potential. It must be considered that species-specific differences, as well as different EV isolation approaches used in each investigation, will contribute to variations and affect comparisons between studies.

Overall, limited research has been conducted on the therapeutical potential of EVs derived from fish, such as the Atlantic cod, while significant research has been published on their unique immune properties [43,44,45,46,47,48,49,50,51,52,53,54,55,56,57,58,59,60]. Importantly, Atlantic cod-derived products—including cod skin collagen [4], cod liver oil [6], and skin xenografts from cod [7]—have shown promising results in tissue regeneration, which highlights beneficial use of cod-derived biomaterials with minimal risk for immunogenicity in clinical applications [7,61,62,63,64]. However, to date, no studies have explored the regenerative potential of EVs from cod biofluids.

Cod serum and mucus EVs were recently described by our group, including with respect to post-translationally deiminated protein cargoes and the export of complement components and CRP, providing new insights into roles in immune defence [10,11,12]. Further functional studies on cod EVs, including in the context of tissue regeneration and wound healing, are therefore warranted. The current pilot study explored for the first time the potential of cod serum- and mucus-derived EV in promoting wound healing using in vitro human and mouse cell lines. The full proteome of cod serum EVs and mucus EVs was also analysed to identify proteins and associated functional pathways which may contribute to putative pro-regenerative functions.

There were significant differences observed between EV protein cargoes of mucus EVs and serum EVs with respect to numbers of and differences in protein hits and associated functional pathway enrichment analysis. There were 49 hits unique to the mucus EV proteome and 15 hits unique to the serum EV proteome. In addition, eight protein hits were shared between the cod serum EVs and mucus EVs: fast skeletal muscle alpha-actin, beta-actin, serotransferrin, 60 S ribosomal protein L22, ribosomal protein L15, galectin, elongation factor 1, alpha and profilin. These have all been linked to roles in mucosal and innate immunity, the acute phase response, apoptosis, and various pathological processes as well as wound healing [65,66,67,68,69,70,71,72,73]. This highlights some shared properties between the cod serum EVs and mucus EVs. Protein–protein interaction network analysis for the cod serum EV and mucus EV proteomes highlighted considerable differences in enriched functional pathways associated with the respective EV proteomes. There was only 1 KEGG pathway associated with the serum EV proteome (platelet activation), while 53 KEGG pathways were associated with the mucus EV proteome. These were linked to bacterial and viral infection; immunodeficiency; inflammatory pathways including IL-17, Toll-like receptor, and TNF signalling; innate immune responses; antigen processing and presentation; phagosomes; allograft rejection; HIF-1 signalling; MAPK signalling; and various autoimmune diseases. This highlights roles for cod mucus EVs in a wide range of immune responses as well as responses to stress and involvement in wound healing pathways [74,75,76,77,78]. Furthermore, 19 reactome pathways were associated with the serum EVs (but none with the mucus EVs), and these were associated with BRAF and RAF signalling, the MAPK pathway, platelet degranulation, hemostasis, TLR2/4 pathways, fibrin clot formation, ROBO receptor signalling, and protein metabolism. This highlights various associations of cod serum EVs with cellular signalling, cell differentiation, responses to stress and angiogenesis, and links to wound healing mechanisms [79,80,81,82,83].

The findings from the cod EV proteome analysis may inform some of the findings from the scratch assay analysis, mimicking wound healing in fibroblast and keratinocyte cell lines in vitro. It must though be noted that a direct comparison of cod serum EV and mucus EV application was not carried out for all three cell lines and all time points. When assessing the potential of cod EVs for regenerative efficacy in vitro, the effects of the cod serum EVs and mucus EVs varied between cell lines but indicated some pro-regenerative potential. The mouse fibroblasts (3T3-L1) showed a trend in wound gap reduction after 24 h treatment with cod serum-derived EVs, while no significant effects were observed for the HaCaT human keratinocyte cell line in response to cod serum EV treatment. The human dermal fibroblast cells (HDFa) showed some promising results with a significantly reduced wound gap closure after 6 h treatment with cod mucus EVs, while significant differences in gap closure were not observed following application with cod serum EVs following 24 h treatment. Furthermore, the application of cod serum EVs and mucus EVs on HDFa cells following scratch injury indicated differing changes in cell expression levels of vimentin and FGF-2, which are crucial regulators of wound-healing processes [84,85]. In the HDFa cells, the serum EVs showed effects on increasing FGF-2 levels, while mucus EV application did not appear to affect FGF-2 levels but did increase vimentin levels, suggesting some differing roles for both types of cod EVs in enhancing wound-healing processes by upregulating these proteins. The FGF family is important for regulating wound healing, with FGF-2 playing roles in stimulating collagen synthesis, epithelialisation, fibronectin, and proteoglycan synthesis. FGFs contribute to the proliferation and/or migration of essential cell types involved in the wound-healing process both in vitro and in vivo, such as fibroblasts and keratinocytes [80]. The potential therapeutic use of FGF-2 in next-generation wound-healing biomaterials has recently been explored [86]. Vimentin belongs to the extensive family of intermediate filaments and has been identified as a highly adaptable cytoskeletal protein crucially engaged in various essential aspects of wound healing. Its significant role in epithelial–mesenchymal transition during wound healing is well established [81]. Furthermore, vimentin actively contributes to diverse cellular processes, including growth, proliferation, migration, cell survival, and stress resilience. As a result, vimentin plays a participatory role in all phases of the wound-healing process [85].

It is important to note that this pilot study forms the basis of more extensive investigations into the roles of EVs derived from cod biological fluids, including serum and mucus EVs. It can be postulated that compared to human-derived EVs, cod biofluid EVs may offer a sustainable product, particularly if they can be processed as a byproduct from the fishing and aquaculture industry. Future studies will need to address the functional pathways of cod EVs in wound healing in more depth in relation to their regenerative capacity. This includes larger sample sizes for the wound healing assays (as the current study used a minimal approach of n = 3) as well as the assessment of a wider range of EV doses on different cellular models and a wider range of time points. In addition, while the current study used a pool of ten samples for individual serum and mucus EV preparations, respectively, future studies will need to assess potential variability both in EV content as well as EV’s efficacy in promoting wound healing between EV preparations from the same biofluid source. This includes the identification of an optimal sample pool size to ensure consistent results and EV yield between EV batches, particularly in the context of standardisation and reproducibility for clinical applications. Scalable production will furthermore be critical for industrial bioprocessing [87]. Moreover, the assessment of in vitro toxicity, cell viability, and cellular uptake of cod EVs will provide further insights into their biological activity in the wound-healing process. While the proteome analysis of the cod EVs revealed different profiles between the two biological fluids, both showed profiles of immune and wound-healing pathways, which may affect different aspects of wound healing. Further evaluation through functional assays, including pathway-specific inhibitors or gene expression analysis, will be valuable in future studies. It must also be noted that the functional in vitro experiments were carried out using archived cod serum and mucus samples, which may have reduced their bioactivity in the wound healing applications in the current study. It will be of interest to further assess cod EV stability, including the effects of freezing–thawing and longer-term storage, all of which will be critical for practical applications. Therefore, while the data shows promise, this pilot study provides only preliminary insights into the potential of cod-derived EVs in wound healing applications.

## 5. Conclusions

This pilot study assessed the proteomic cargo content of extracellular vesicles (EVs) from cod serum and mucus and assessed their in vitro potential for regenerative activity in wound-healing processes using fibroblast and keratinocyte cellular models. A pro-regenerative potential of cod serum EVs and mucus EVs was identified, also showing some differences between fibroblast and keratinocyte cell lines. This was further confirmed by the varying effects of the cod serum EVs and mucus EVs on elevating vimentin and FGF-2 levels, following EV application to the in vitro cellular scratch injury models. The assessment of the cod EVs’ protein cargoes associated with immune, stress, and wound-healing pathways provides valuable insights for future in-depth studies on the mechanisms involved. EVs isolated from Atlantic cod biofluids, including mucus and serum, may represent sustainable innovative therapeutic options for regenerative medicine applications.

## Data Availability

All the data is provided within the article and Supplementary Materials.

## Supplementary Materials

The following supporting information can be downloaded at: https://www.mdpi.com/article/10.3390/biology14070870/s1, Table S1: The cod serum EV proteome. Total protein hits of cod serum EV cargoes, as identified by LC-MS/MS analysis. Protein ID, protein name, matches (sequences), and total score are shown. Highlighted protein hits were identified as common to the cod serum EV and cod mucus EV proteomes. Table S2: The cod mucus EV proteome. Total protein hits of cod mucus EV cargoes, as identified by LC-MS/MS analysis. Protein ID, protein name, matches (sequences) and total score are shown. Highlighted protein hits are hits identified as common to the cod serum EV and cod mucus EV proteomes.
