# Supplementary material for: Exploring the Potential of Extracellular Vesicles from Atlantic Cod (Gadus morhua L.) Serum and Mucus for Wound Healing In Vitro
Source: Biology (Basel). 2025 Jul 17;14(7):870. doi: 10.3390/biology14070870 (PMC12292778; doi:10.3390/biology14070870)
Supplement: Supplementary file 1 [file biology-14-00870-s001.zip › biology-3754973-supplementary.pdf]

Supplementary Table S1. The cod serum-EV proteome. Total protein hits of cod serum-EV cargoes, as identified by LC-MS/MS analysis. Protein ID, protein name, matches (sequences) and total score are shown. Highlighted protein hits were identified as common to the cod serum-EV and cod mucus-EV proteomes.

**Protein ID**<br>**Protein name**	***Species name***	**Matches**<br>**(Sequences)**	**Total score**<br>**($p < 0.05$)[†]**
Q78AY8_GADMO<br>Fast skeletal muscle alpha-actin	*Gadus morhua*	17<br>(5)	214
Q2PDJ0_GADMO<br>Beta-actin	*Gadus morhua*	7<br>(4)	138
Q92079｜TRFE_GADMO<br>Serotransferrin	*Gadus morhua*	17<br>(2)	106
P52865｜RL22_GADMO<br>60S ribosomal protein L22	*Gadus morhua*	1<br>(1)	65
G8ENP0_GADMO<br>Galectin	*Gadus morhua*	1<br>(1)	40
A8CZC9_GADMO<br>Elongation factor 1 alpha	*Gadus morhua*	3<br>(1)	40
A0A067XLH1_GADMO<br>Profilin	*Gadus morhua*	3<br>(1)	35
Q8JHA8_GADMO<br>Ribosomal protein L15	*Gadus morhua*	1<br>(1)	21
A0A498LWK8_LABRO<br>Intermediate filament ON3-like isoform X2	*Labeo rohita*	55<br>(7)	293
A0A3Q2UKY4_FUNHE<br>IF rod domain-containing protein	*Fundulus heteroclitus*	48<br>(7)	283
A0A0P7W925_SCLFO<br>Histone H2A	*Scleropages Formosus*	8<br>(4)	224
A0A6I9NC47_9TELE<br>fibrinogen beta chain	*Notothenia coriiceps*	16<br>(3)	135
A0A3B4DU27_PYGNA<br>Fibrinopeptide A	*Pygocentrus nattereri*	9<br>(2)	79
A0A0P7UK88_SCLFO<br>Elastase 3-like	*Scleropages Formosus*	14<br>(1)	69
A0A060VVQ0_ONCMY<br>14_3_3 domain-containing protein	*Oncorhynchus mykiss*	2<br>(2)	69
A0A0P7VZ44_SCLFO<br>Glyceraldehyde-3-phosphate dehydrogenase	*Scleropages formosus*	3<br>(2)	69
A0A3B4AU77_9GOBI<br>ATP synthase lipid-binding protein	*Periophthalmus magnuspinnatus*	4<br>(2)	63
A0A3B4APH2_9GOBI<br>Integrin_alpha2 domain-containing protein	*Periophthalmus magnuspinnatus*	2<br>(2)	62
A0A4W6F2L3_LATCA<br>Matrilin 2	*Lates calcarifer*	2<br>(2)	61
A0A3P8SBB8_AMPPE<br>Apolipoprotein Ba	*Amphiprion percula*	11<br>(2)	60
A0A087XS38_POEFO<br>TsaA-like domain-containing protein	*Poecilia formosa*	8<br>(1)	60
A0A087XBK7_POEFO<br>Deacetylase sirtuin-type domain-containing protein	*Poecilia formosa*	4<br>(2)	54
A0A060XZN5_ONCMY<br>Sulfotransferase	*Oncorhynchus mykiss*	4<br>(2)	54

[†] Ion score is −10*Log(P), where P is the probability that the observed match is a random event. Individual ion scores >16 indicate identity or extensive similarity ($p < 0.05$) for the cod database. When compared to the teleost database, individual ions scores cut-off were set at > 53. Protein scores are derived from ion scores as a non-probabilistic basis for ranking protein hits.

Supplementary Table S2. The cod mucus-EV proteome. Total protein hits of cod mucus-EV cargoes, as identified by LC-MS/MS analysis. Protein ID, protein name, matches (sequences) and total score are shown. Highlighted protein hits are hits identified as common to the cod serum-EV and cod mucus-EV proteomes.

**Protein ID**<br>**Protein name**	***Species name***	**Matches**<br>**(Sequences)**	**Total score**<br>**($p < 0.05$)[I]**
Q92079∣TRFE_GADMO<br>Serotransferrin	*Gadus morhua*	32<br>(28)	1747
V9I305∣V9I305_GADMO<br>Transglutaminase 2	*Gadus morhua*	26<br>(18)	1086
G8DZS1_GADMO<br>Heat shock cognate 70 kDa protein	*Gadus morhua*	29<br>(14)	1081
Q9PUG4_GADMO<br>Tubulin beta chain	*Gadus morhua*	30<br>(16)	996
A8CZC9_GADMO<br>Elongation factor 1-alpha	*Gadus morhua*	25<br>(14)	805
V9I378_GADMO<br>Transglutaminase 1	*Gadus morhua*	18<br>(13)	757
A0A067XL91_GADMO<br>Flotillin-1	*Gadus morhua*	11<br>(9)	616
K7SPU9_GADMO<br>MHC class I antigen	*Gadus morhua*	13<br>(10)	599
A0A067XL41_GADMO<br>Calpain small subunit 1	*Gadus morhua*	13<br>(11)	577
Q78AY8_GADMO<br>Fast skeletal muscle alpha-actin	*Gadus morhua*	58<br>(9)	565
P56533∣BADH_GADMC<br>Betaine aldehyde dehydrogenase	*Gadus morhua*	11<br>(11)	540
K7S1G8_GADMO<br>MHC class I antigen	*Gadus morhua*	12<br>(9)	532
Q2PDJ0_GADMO<br>Beta-actin	*Gadus morhua*	58<br>(8)	511
Q6WEU6_GADMO<br>S2 ribosomal protein	*Gadus morhua*	9<br>(8)	467
Q8JHA8_GADMO<br>Ribosomal protein L15	*Gadus morhua*	11<br>(7)	419
G8DZS2_GADMO<br>Nucleoside diphosphate kinase	*Gadus morhua*	10<br>(6)	388
G0XNX4_GADMO<br>Peptidyl-prolyl cis-trans isomerase	*Gadus morhua*	7<br>(6)	331
P83456∣PPB_GADMO<br>Alkaline phosphatase	*Gadus morhua*	8<br>(8)	307
Q5XQS6_GADMO<br>Preproapolipoprotein A-I	*Gadus morhua*	5<br>(4)	305
A0A067XLH1_GADMO<br>Profilin	*Gadus morhua*	7<br>(4)	249
A0A0G2QMS5_GADMO<br>Histone H3	*Gadus morhua*	31<br>(3)	205
D5LIQ8_GADMO<br>Putative ribosomal protein L8	*Gadus morhua*	6<br>(4)	201
G0XNX5_GADMO<br>Cystatin B	*Gadus morhua*	9<br>(5)	201
A8CZB9_GADMO<br>20-beta hydroxysteroid dehydrogenase	*Gadus morhua*	4<br>(4)	197
P81600∣ADHH_GADMO<br>Alcohol dehydrogenase class-3 chain H	*Gadus morhua*	3<br>(3)	184
P52865∣RL22_GADMO<br>60S ribosomal protein L22	*Gadus morhua*	4<br>(2)	182
G0XNX6_GADMO<br>Mannan-binding lectin	*Gadus morhua*	4<br>(4)	169
A0A343ANK4_GADMO<br>Cytochrome c oxidase subunit 2	*Gadus morhua*	4<br>(3)	142

D5LIQ2_GADMO<br>Pantophysin	*Gadus morhua*	2<br>(2)	138
G8ENP0_GADMO<br>Galectin	*Gadus morhua*	13<br>(2)	136
E3U9P6_GADMO<br>Bloodthirsty	*Gadus morhua*	3<br>(3)	117
Q8AWX8_GADMO<br>Glyceraldehyde-3-phosphate dehydrogenase	*Gadus morhua*	3<br>(2)	115
G0XNX7_GADMO<br>Peptidylprolyl isomerase	*Gadus morhua*	2<br>(2)	108
D5LIQ1_GADMO<br>Putative ribosomal protein L17b	*Gadus morhua*	3<br>(2)	106
A7XA14_GADMO<br>Nascent polypeptide-associated complex alpha polypeptide NACA	*Gadus morhua*	1<br>(1)	74
Q9YGI3_GADMO<br>Beta-2-microglobulin	*Gadus morhua*	2<br>(1)	66
K9LCP7_GADMO<br>Caspase 3	*Gadus morhua*	1<br>(1)	55
D7R9W9_GADMO<br>Fas	*Gadus morhua*	1<br>(1)	44
D7R9Z6_GADMO<br>Mitogen-activated protein kinase 1	*Gadus morhua*	2<br>(1)	42
D7R9Y5_GADMO<br>Toll-like receptor 9	*Gadus morhua*	1<br>(1)	41
Q6DTY9_GADMO<br>Glucose transporter 3	*Gadus morhua*	1<br>(1)	34
A7XA12_GADMO<br>Aminopeptidase puromycin-sensitive protein	*Gadus morhua*	1<br>(1)	27
A4ZGE0_GADMO<br>Non-specific cytotoxic cell receptor protein 1	*Gadus morhua*	1<br>(1)	24
B8XJS4_GADMO<br>Hemoglobin alpha 1 chain	*Gadus morhua*	1<br>(1)	24
Q9PV18_GADMO<br>RAG1 protein	*Gadus morhua*	1<br>(1)	24
D6MXZ1_GADMO<br>Acyl-Coenzyme A dehydrogenase	*Gadus morhua*	1<br>(1)	24
Q8JIV4_GADMO<br>Fast skeletal myosin heavy chain	*Gadus morhua*	1<br>(1)	23
G8DZS4_GADMO<br>Hypoxanthine phosphoribosyltransferase	*Gadus morhua*	1<br>(1)	23
A0A0D3RBU4_GADMO<br>Interferon regulatory factor protein 7	*Gadus morhua*	1<br>(1)	23
A7XA16_GADMO<br>Titin isoform b	*Gadus morhua*	1<br>(1)	23
F8SXV2_GADMO<br>Osteonectin	*Gadus morhua*	(1)	23
A7XA17_GADMO<br>Titin isoform c	*Gadus morhua*	1<br>(1)	22
Q1M164_GADMO<br>Interleukin-8	*Gadus morhua*	1<br>(1)	22
F8TW94_GADMO<br>Activating transcription factor 3	*Gadus morhua*	1<br>(1)	22
A0A0C6EYL8_GADMO<br>Amine oxidase	*Gadus morhua*	1<br>(1)	22
D7R9Z7_GADMO<br>Mitogen-activated protein kinase	*Gadus morhua*	1<br>(1)	21
A7UFK3_GADMO<br>Hexokinase 1a	*Gadus morhua*	1<br>(1)	21

† Ion score is −10*Log(P), where P is the probability that the observed match is a random event. Individual ion scores > 16 indicate identity or extensive similarity ($p < 0.05$) for the cod database. Protein scores are derived from ion scores as a non-probabilistic basis for ranking protein hits.
