# Supplementary figures and images for: Exploring the Potential of Extracellular Vesicles from Atlantic Cod (Gadus morhua L.) Serum and Mucus for Wound Healing In Vitro
Source: Biology (Basel). 2025 Jul 17;14(7):870. doi: 10.3390/biology14070870 (PMC12292778; doi:10.3390/biology14070870)

Figure 1 C

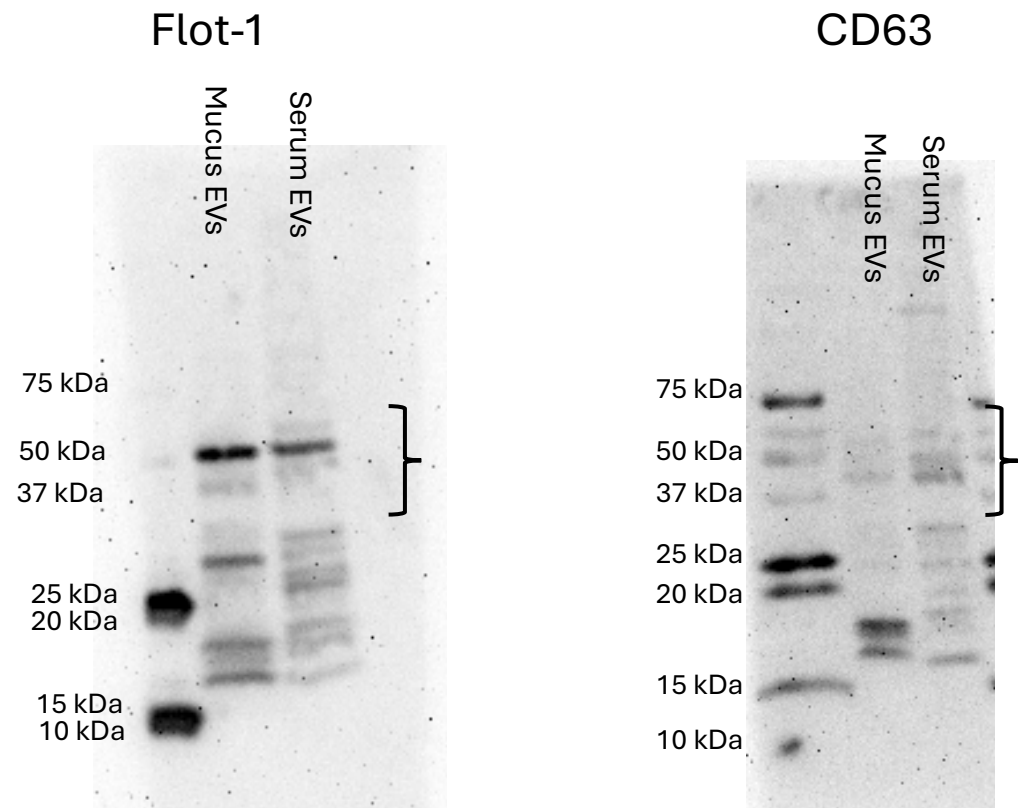

Supplement: Supplementary file 1 [file biology-14-00870-s001.zip › Figure S1. Full blots cod EVs.pdf]
